# The *MC4R* p.Ile269Asn mutation confers a high risk for type 2 diabetes in the Mexican population via obesity dependent and independent effects

**DOI:** 10.1038/s41598-021-82728-w

**Published:** 2021-02-04

**Authors:** Miguel Vázquez-Moreno, Daniel Locia-Morales, Adan Valladares-Salgado, Tanmay Sharma, Aleyda Perez-Herrera, Roxana Gonzalez-Dzib, Francisco Rodríguez-Ruíz, Niels Wacher-Rodarte, Miguel Cruz, David Meyre

**Affiliations:** 1 Unidad de Investigación Médica en Bioquímica, Hospital de Especialidades, Centro Médico Nacional Siglo XXI, Instituto Mexicano del Seguro Social, Av. Cuauhtémoc, 330, C.P. 06725 Mexico City, Mexico; 2 Department of Health Research Methods, Evidence, and Impact, Michael DeGroote Centre for Learning and Discovery, McMaster University, Room 3205, 1280 Main Street West, Hamilton, ON L8S 4K1 Canada; 3 Consejo Nacional de Ciencia y Tecnología, Instituto Politécnico Nacional-Centro Interdisciplinario de Investigación para el Desarrollo Integral-Regional Unidad Oaxaca, Oaxaca City, Mexico; 4 Instituto Mexicano del Seguro Social, Campeche, Mexico; 5 Unidad de Investigación en Epidemiología Clínica, Hospital de Especialidades, Centro Médico Nacional Siglo XXI, Instituto Mexicano del Seguro Social, Mexico City, Mexico; 6 Department of Pathology and Molecular Medicine, McMaster University, Hamilton, Canada

**Keywords:** Diabetes, Obesity, Genetics

## Abstract

We investigated the association between the loss-of-function mutation *MC4R* p.Ile269Asn and T2D risk in the Mexican population. We enrolled 6929 adults [3175 T2D cases and 3754 normal glucose tolerant (NGT) controls] and 994 NGT children in the study. Anthropometric data and T2D-related quantitative traits were studied in 994 NGT children and 3754 NGT adults. The *MC4R* p.Ile269Asn mutation was genotyped using TaqMan. The *MC4R* p.Ile269Asn mutation was associated with T2D [OR = 2.00, 95% confidence interval (CI) 1.35–2.97, *p* = 0.00057] in Mexican adults. Additional adjustment for body-mass index (BMI) attenuated but did not remove the association (OR = 1.70, 95% CI 1.13–2.56, *p* = 0.011). The *MC4R* p.Ile269Asn mutation was associated with T2D (OR = 1.88, 95% CI 1.14–3.08, *p* = 0.013) in a subset of 1269 T2D cases and 1269 NGT controls matched for sex, age, and BMI. A mediation analysis estimated that BMI accounts for 22.7% of the association between *MC4R* p.Ile269Asn mutation and T2D risk (*p* = 4.55 × 10^–6^). An association was observed between the *MC4R* p.Ile269Asn mutation and BMI in NGT children and adults (children: beta = 3.731 ± 0.958, *p* = 0.0001; adults: beta = 2.269 ± 0.536, *p* = 2.3 × 10^–5^). In contrast, the mutation was not associated with T2D-related quantitative traits. We demonstrate that the *MC4R* p.Ile269Asn mutation predisposes to T2D via obesity-dependent and independent effects in the Mexican population.

## Introduction

In the last 5 decades, type 2 diabetes (T2D) and obesity rates have increased in parallel and reached epidemic proportions. According to the World Health Organization (WHO), 650 million individuals were obese, and 422 million had diabetes in 2015–2016. Obesity rise has been established as a driving factor for the current T2D epidemic^1^. Both diseases result from the interplay of environmental and biological factors, including genetics^2^. Body mass index (BMI) and T2D share one-fifth of genetic variance^3^. Genome-wide association studies have identified common loci contributing to obesity and T2D^2^. Several polygenic variants (e.g., *FTO*, *MC4R, TMEM18, SEIC6B*) increase T2D risk mediated by their obesity-predisposing effects in populations of European ancestry^2,4^. However, little is known about the contribution of rare variants predisposing to oligogenic/monogenic obesity to T2D. Furthermore, few studies have investigated the high-obesity / T2D risk Mexican population within this context^5^. In 2019, a study identified a significant exome-wide association between the p.Ile269Asn/rs79783591 A > T mutation in the melanocortin-4 receptor (*MC4R*) gene and T2D in Hispanic/Latino individuals (minor allele frequency (MAF): 0.89%, OR = 2.17, 95% confidence interval 1.63–2.89, *p* = 4 × 10^–7^)^6^. The authors mentioned that conditioning on BMI reduced but did not eliminate the *MC4R* p.Ile269Asn T2D association (*p* = 1 × 10^–5^)^6^. Unfortunately, they did not report OR for the BMI-adjusted test, which makes the interpretation of the results difficult^6^. The *MC4R* p.Ile269Asn mutation has not been found in individuals of African, East Asian, South Asian, and European ancestry^7^. While the *MC4R* p.Ile269Asn mutation is absent from most Hispanic/Latino populations (Columbians, Peruvians, and Puerto Ricans), it is polymorphic in the Mexican population^7^. Collectively, these data suggest that the mutation emerged as a founder mutation in the native Mexican population^7^. In vitro functional experiments have previously demonstrated that the p.Ile269Asn mutation reduces cell surface expression of the MC4R and impairs its ability to bind alpha-MSH and induce cAMP production in response to the agonist^8–10^. The leptin-melanocortin axis controls human energy balance, and the MC4R is a key player in its central regulation^11^. While MC4R partial and complete deficiency results in hyperphagia and oligogenic/monogenic obesity in mice and humans^12,13^, there is limited evidence in the literature linking MC4R to T2D^9,14^. This prompted us to investigate whether the *MC4R* p.Ile269Asn mutation increases T2D risk via obesity-dependent or independent mechanisms in the Mexican population.

## Results

### Association of the *MC4R* p.Ile269Asn mutation with T2D in Mexican adults

We investigated the association between the *MC4R* p.Ile269Asn mutation and T2D in 3175 and 3754 Mexican adults with T2D and NGT, respectively. We had an excellent statistical power to detect the genetic effect previously reported in literature in our study design (99.9% chance to detect an OR of 2.2, Supplementary Table 1). Anthropometric and genetic data are presented in Table 1. When compared to the NGT group, the T2D group included more women (43.1% vs 35.6%) and displayed higher age (55.9 ± 9.5 *versus* 43.4 ± 8.1 years), BMI (29.5 ± 4.8 vs 28.0 ± 4.2 kg/m^2^), fasting plasma glucose (FPG, 7.0 ± 3.1 vs 4.5 ± 0.5 mmol/l), and hemoglobin A1c (HbA1c, 6.2 ± 1.1 vs 5.3 ± 0.3%). The mutation was 49% more prevalent in participants with T2D than with NGT (MAF = 1.21% vs 0.81%) and was positively associated with T2D under an additive model (OR = 2.00, 95% CI [1.35–2.97], *p* = 0.00057, adjustments for age and sex, Table 2). Additional adjustment for BMI attenuated but did not remove the association (OR = 1.70, 95% CI [1.13–2.56], *p* = 0.011, Table 2). To exclude the possibility of spurious association caused by population stratification, we then tested the association between the *MC4R* p.Ile269Asn mutation and T2D before and after adjustment for NAM, EUR, and AFR ancestry proportion in a subset of 688 and 386 adults with NGT and T2D for whom genome-wide SNP genotyping data were available. We had an adequate statistical power to detect the genetic effect previously reported in literature in this subsample (84.3% chance to detect an OR of 2.2, Supplementary Table 1). General characteristics of Mexican adults with and without genome-wide SNP genotyping data are presented in Supplementary Table 2. BMI and frequency of T2D were significantly lower in adults with genome-wide genotyped data, while age was significantly higher. Sex ratio and the frequency of T allele and the genotypes of *MC4R* rs79783591 (A/A, A/T, TT) were not significantly different in the genome-wide genotyped and other adults. Although the association between *MC4R* p.Ile269Asn mutation and T2D was not significant in this subset of adults, possibly as a consequence of a Winner’s curse effect (overestimation of a true genetic effect in the original report)^6,15^, the OR estimates did not sensibly change before (OR = 1.95, 95% CI [0.69–5.48], *p* = 0.207, model adjusted for age and sex/OR = 1.63, 95% CI [0.56–4.75], *p* = 0.373, model adjusted for age, sex and BMI) and after adjustment for population stratification (OR = 1.93, 95% CI [0.69–5.39], *p* = 0.208, model adjusted for age, sex and, NAM, EUR and AFR ancestry proportions/OR = 1.64, 95% CI [0.57–4.76], *p* = 0.360, model adjusted for age, sex, BMI and, NAM, EUR and AFR ancestry proportions). Altogether, these findings represent an independent replication of the association between the *MC4R* p.Ile269Asn mutation and T2D before and after adjustment for BMI in the Mexican population, originally reported by Flannick et al. in Hispanic/Latino individuals^6^.

**Table 1:** General characteristics of Mexican adults with NGT and T2D.

Trait	NGT	T2D	*p* value
	N = 3754	N = 3175	–
Women, n (%)	1336 (35.6)	1368 (43.1)	**1.3 × 10**^**–13**^
Age, (years)	43.4 ± 8.1	55.9 ± 9.5	**1.9 × 10**^**–16**^
BMI, (kg/m^2^)	28.0 ± 4.2	29.5 ± 4.8	**1.0 × 10**^**–13**^
FPG (mmol/l)^a^	4.5 ± 0.5	7.0 ± 3.1	**8.6 × 10**^**–41**^
HbA1c (%)^b^	5.3 ± 0.3	6.2 ± 1.1	**1.0 × 10**^**–24**^
Rs79783591 T allele frequency, (%)	0.81	1.21	**0.018**
Rs79783591 A/A, n (%)	3 694 (98.40)	3 099 (97.61)	0.060
Rs79783591 A/T, n (%)	59 (1.57)	75 (2.36)	
Rs79783591 T/T, n (%)	1 (0.027)	1 (0.031)	

Data are expressed as mean ± standard deviation and N (%). NGT, normal glucose tolerance; T2D, type 2 diabetes; BMI, body mass index; FPG, fasting plasma glucose; HbA1c, hemoglobin A1c. The difference in sex ratios was analyzed using the X^2^ test. Differences in means were analyzed using Student's t-tests. Significant *p* values (*p* < 0.05) are reported in bold. ^a^FPG values were available in 3754 NGT and 376 T2D participants.

^b^HbA1c values were available in 1620 NGT and 246 T2D participants.

**Table 2:** Association between the *MC4R* p.Ile269Asn mutation and type 2 diabetes in Mexican adults using conventional and matched case–control study designs.

**No adjustment**	**N**_**T2D**_	**N**_**NGT**_	**OR, [95% confidence interval] (p value)**
*MC4R* p.Ile269Asn (unmatched case control)	3175	3754	–
*MC4R* p.Ile269Asn (age-, sex-, and BMI-matched case control)	1269	1269	1.88, [1.14–3.08] (0.013)
**Adjustment for age and sex**	**N**_**T2D**_	**N**_**NGT**_	**OR, 95% confidence interval (p value)**
*MC4R* p.Ile269Asn (unmatched case control)	3175	3754	2.00, [1.35–2.97] (0.00057)
*MC4R* p.Ile269Asn (age-, sex-, and BMI-matched case control)	1269	1269	1.88, [1.14–3.08] (0.0129)
**Adjustment for age, sex and BMI**	**N**_**T2D**_	**N**_**NGT**_	**OR, 95% confidence interval (p value)**
*MC4R* p.Ile269Asn (unmatched case control)	3175	3754	1.70, [1.13–2.56] (0.011)
*MC4R* p.Ile269Asn (age-, sex-, and BMI-matched case control)	1269	1269	1.88, [1.14–3.09] (0.0129)

*T2D* type 2 diabetes, *NGT* normal glucose tolerance, *BMI* body mass index, *OR* odds-ratio.

### Mediation effect of BMI on the association between the *MC4R* p.Ile269Asn mutation and T2D risk

**Figure 1:**
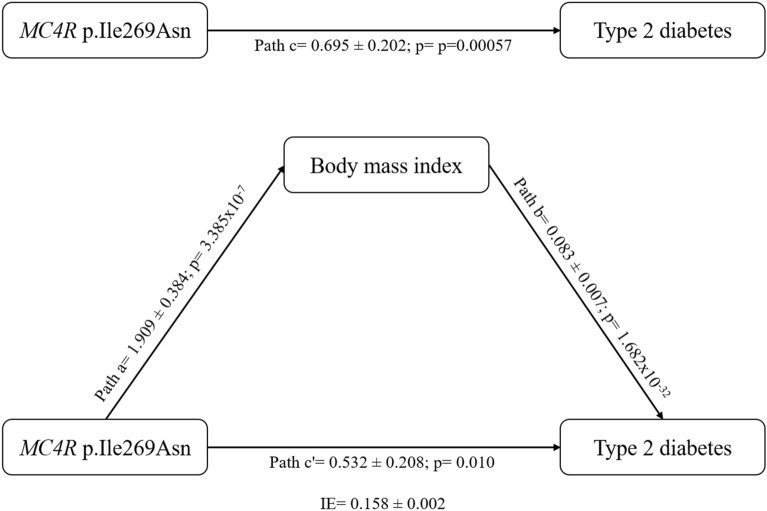
Simple mediation model evaluating the effect of body mass index as a mediator of the association between *MC4R* p.Ile269Asn and type 2 diabetes (% of mediation = 22.7%, Sobel test: z = 4.584; *p* = 4.55 × 10^–6^). Path a, effect of *MC4R* p.Ile269Asn on body mass index (data is expressed as beta value ± standard error analyzed by a linear regression model adjusted for age and sex); Path b, effect of body mass index on type 2 diabetes (data is expressed as Log Odds Ratio ± standard error analyzed by a logistic regression model controlled for *MC4R* p.Ile269Asn, and adjusted for age and sex); Path c, total effect of *MC4R* p.Ile269Asn on type 2 diabetes (data is expressed as Log Odds Ratio ± standard error analyzed by a logistic regression model adjusted for age and sex); Path c′, effect of *MC4R* p.Ile269Asn on type 2 diabetes (data is expressed as Log Odds Ratio ± standard error analyzed by a logistic regression model controlled for body mass index, and adjusted for age and sex); IE, indirect effect of *MC4R* p.Ile269Asn on type 2 diabetes (data is expressed as the product of path a and path b); % of mediation is computed as IE divided by path c.

We used the Sobel test to evaluate BMI’s significant mediation effect on the association between *MC4R* p.Ile269Asn and T2D risk in Mexican adults^16^. Based on Baron and Kenny’s assumptions^17^, we first evaluated the pertinence to use BMI as a mediator for the association between *MC4R* p.Ile269Asn mutation and T2D risk (Fig. 1). Firstly, *MC4R* p.Ile269Asn mutation was positively associated with T2D (OR = 2.00 95% CI [1.35–2.97], *p* = 0.00057, logistic regression model adjusted for age and sex). Secondly, the *MC4R* p.Ile269Asn mutation was positively associated with BMI (β = 1.909 ± 0.384, *p* = 3.4 × 10^–7^, linear regression model adjusted for age and sex). Thirdly, controlling for MC4R p.Ile269Asn, BMI was positively associated with T2D (OR = 1.09, 95% CI [1.07–1.10], *p* = 1.7 × 10^–32^, logistic regression model adjusted for age and sex). Based on these results, we then assessed the mediation effect of BMI on the association between *MC4R* p.Ile269Asn mutation and T2D risk. The Sobel test was significant (z = 4.584; *p* = 4.55 × 10^–6^), and BMI was estimated to mediate 22.7% of the positive association between *MC4R* p.Ile269Asn mutation and T2D risk (test adjusted for age and sex).

### Association of the *MC4R* p.Ile269Asn mutation with T2D in a matched case–control study

We then used a T2D case–control design matched for sex, age, and BMI to investigate whether the association between *MC4R* p.Ile269Asn and T2D in Mexican adults was driven by its BMI-increasing effect. A total of 1269 T2D cases and 1269 NGT controls were matched for sex, age (range ± 0.5 years), and BMI (range ± 0.5 kg/m^2^) from our initial sample. We had an excellent statistical power to detect the genetic effect previously reported in literature in our study design (97.7% chance to detect an OR of 2.2, Supplementary Table 1). Anthropometric and genetic data are presented in Table 3. The *MC4R* p.Ile269Asn mutation was 88% more prevalent in participants with T2D than with NGT (MAF = 1.77% *versus* 0.94%). The *MC4R* p.Ile269Asn variant allele was positively associated with T2D (OR = 1.88, 95% CI [1.14–3.08], *p* = 0.013, Table 2). As expected, further adjustment for age and sex (OR = 1.88, 95% CI [1.14–3.08], *p* = 0.0129), or for age, sex and BMI (OR = 1.88, 95% CI [1.14–3.09], *p* = 0.0129) did not alter the association (Table 2).

**Table 3:** General characteristics of Mexican adults with T2D and NGT matched for sex, age (range ± 0.5 years), and BMI (range ± 0.5 kg/m^2^).

Trait	NGT	T2D	*p* value
	N = 1269	N = 1269	–
Women, N (%)	490 (38.6)	490 (38.6)	1.000
Age (years)	49.30 ± 7.30	49.30 ± 7.30	1.000
BMI (kg/m^2^)	28.7246 ± 3.57756	28.7346 ± 3.58772	0.942
Rs79783591 T allele frequency (%)	0.94	1.77	**0.010**
Rs79783591 A/A, N (%)	1 246 (98.19)	1 224 (96.45)	**0.011**
Rs79783591 A/T, N (%)	22 (1.73)	45 (3.55)	
Rs79783591 T/T, N (%)	1 (0.079)	0 (0)	

Data are expressed as mean ± standard deviation. *NGT* normal glucose tolerance, *T2D* type 2 diabetes, *BMI* body mass index. The difference in sex ratios was analyzed using the X^2^ test. Differences in means were analyzed using Student's t-tests. Significant *p* values (*p* < 0.05) are reported in bold.

### Association of the *MC4R* p.Ile269Asn mutation with T2D-related quantitative traits in Mexican adults with NGT

We investigated whether the *MC4R* p.Ile269Asn mutation was associated with T2D-related quantitative traits (BMI, waist circumference (WC), FPG, 2-h post-oral glucose tolerance test plasma glucose (2-h PG), fasting plasma insulin (FPI), homeostatic model assessment of insulin resistance (HOMA-IR) and beta-cell function (HOMA-B)) in 3 754 Mexican adults with NGT (Table 4, Supplementary Table 3). Participants displayed an average age and BMI of 43.4 ± 8.1 years and 28.0 ± 4.2 kg/m^2^, respectively, and 35.6% were women. We only found a positive association between the mutation and BMI in this population (beta = 2.269 ± 0.536, *p* = 2.3 × 10^–5^).

**Table 4:** Association between the *MC4R* mutation and T2D-related quantitative traits in a Mexican NGT population.

Trait	Adults			Children		
	N	β ± SE (*p* value)^a^	β ± SE (*p* value)^b^	N	β ± SE (*p* value)^c^	β ± SE (*p* value)^d^
BMI	3754	2.269 ± 0.536 (**2.3 × 10**^**–5**^)	NA	994	3.731 ± 0.958 (**0.0001)**	NA
BMI-SDS	NA	NA	NA	994	0.646 ± 0.235 (**0.006)**	NA
WC (cm)	1266	0.124 ± 1.926 (0.946)	− 2.021 ± 1.305 (0.122)	919	7.4 ± 2.5 **(0.003)**	2.1 ± 1.7 (0.229)
FPG (mmol/L)	3754	− 0.128 ± 0.126 (0.309)	− 0.030 ± 0.124 (0.811)	994	− 0.183 ± 0.108 (0.089)	− 0.180 ± 108 (0.096)
2-h PG (mmol/L)	1619	0.154 ± 0.387 (0.691)	− 0.094 ± 0.385 (0.808)	NA	NA	NA
FPI (µU/mL)	960	0.268 ± 1.543 (0.862)	0.453 ± 1.542 (0.769)	570	1.786 ± 1.497 (0.233)	1.059 ± 1.370 (0.440)
HOMA-IR	960	0.378 ± 0.423 (0.372)	0.091 ± 0.382 (0.812)	570	0.375 ± 0.318 (0.239)	0.224 ± 0.292 (0.444)
HOMA-B	960	16.846 ± 20.025 (0.400)	6.249 ± 18.903 (0.741)	570	41.9 ± 30.7 (0.172)	29.5 ± 28.606 (0.302)

*BMI* body mass index, *BMI-SDS* age- and sex-adjusted standard deviation scores of BMI, *WC* waist circumference, *FPG* fasting plasma glucose, *2-h PG* 2-h plasma glucose, *FPI* fasting plasma insulin, *HOMA-IR* homeostatic model assessment of insulin resistance. *HOMA-B* homeostasis model assessment of beta-cell function, *NA* not analyzed. Linear regression models were adjusted for (a) age and sex, (b) age, sex, and BMI, (c) age, sex, and Mexico state, (d) age, sex, Mexico state, and BMI-SDS. Significant *p* values (*p* < 0.007 after Bonferroni correction [0.05/7]) are reported in bold.

### Association of the *MC4R* p.Ile269Asn mutation with T2D-related quantitative traits in Mexican children with NGT

We then investigated whether the *MC4R* p.Ile269Asn mutation was associated with T2D-related quantitative traits (BMI, BMI-SDS, WC, FPG, FPI, HOMA-IR, HOMA-B) in an independent sample of 994 children with NGT (Table 4, Supplementary Table 3). Children displayed an average age, BMI, and BMI-SDS of 8.9 ± 1.9 years, 16.6 ± 4.3 kg/m^2^, and 0.78 ± 1.28 units, respectively, and 50.5% were girls. We found a positive association between the mutation, BMI, and BMI-SDS in this population (beta = 3.731 ± 0.958, *p* = 0.0001 and beta = 0.646 ± 0.235, *p* = 0.006, respectively). In addition, we found a positive association between the mutation and WC (beta = 7.4 ± 2.5, *p* = 0.003), that did not remain significant after the test was adjusted for BMI-SDS (beta = 2.1 ± 1.7, *p* = 0.229). This means that the association between the *MC4R* p.Ile269Asn mutation and WC in children is not independent from its effect on BMI-SDS.

## Discussion

This study provides compelling evidence that the *MC4R* p.Ile269Asn mutation confers high-risk for T2D via obesity-dependent and independent mechanisms in the Mexican population. Adjusting a conventional T2D case–control association test for BMI attenuated but did not remove the effect of *MC4R* p.Ile269Asn on T2D. Concurrently, a T2D association test, including cases and controls matched for sex, age, and BMI, was significant. A mediation analysis confirmed that the association between the *MC4R* p.Ile269Asn mutation and T2D was not only explained by its obesity-predisposing effect. Indeed, we estimated that BMI only mediated 22.7% of the association between *MC4R* p.Ile269Asn mutation and T2D risk. A consistent association was observed between *MC4R* p.Ile269Asn and BMI in children and adults with NGT. However, the mutation was not associated with conventional T2D-related traits such as FPG, FPI, HOMA-IR, or HOMA-B.

Flannick et al*.* identified a significant association between the *MC4R* p.Ile269Asn mutation and T2D in Hispanic/Latino individuals, which was reduced but not eliminated by adjustment for BMI^6^. We confirmed the findings by Flannick et al*.* in Hispanic/Latino individuals in an independent and homogeneous sample of Mexican adults living in Mexico City. While partial/complete loss-of-function mutations in *MC4R*, including the partial loss-of-function p.Ile269Asn mutation, have been consistently shown to increase BMI / obesity risk in diverse populations^7,9,13,18^, BMI-dependent and independent associations with elevated FPI have been reported in some but not all cohorts, including ours^9,18–20^. Whereas both male and female mice partially or fully deficient in Mc4r are hyperinsulinemic, only males heterozygous or homozygous for a deletion in *Mc4r* develop hyperglycemia^12^. The onset of hyperglycemia in adult Mc4r deficient mice is mainly explained by obesity-induced insulin resistance^21^. On the other hand, it is well-established that the MC4R is involved in glycemic control, independent of its body weight effects. Central Mc4r blockade abolishes the central nervous system-mediated antidiabetic actions of leptin in mice^22^. Mc4r rescue in the lateral hypothalamus improves sympathetic nervous system-dependent glucose tolerance in mice without altering body weight^21^. The MC4R is also expressed in enteroendocrine L cells and regulates the release of peptide YY and glucagon-like peptide 1 (GLP-1) in mice and humans^14^. Deletion of Mc4r genes in both extra-hypothalamic sympathetic and parasympathetic cholinergic neurons impairs glucose homeostasis in mice^23^. Mc4r-deficient mice show exaggerated circadian fluctuations in baseline blood glucose and glucose tolerance. Interestingly, exposure to lighting conditions that disrupt circadian rhythms ameliorates their glucose tolerance. This improvement occurs through an increase in glucose clearance by skeletal muscle and is food intake and body weight independent^24^. We could not find an association with T2D-related traits such as FPG, FPI, HOMA-IR, or HOMA-B in Mexican children and adults with NGT. An obvious explanation may be the modest statistical power of our study (Supplementary Table 4, Supplementary Table 5). Alternatively, the mutation may be associated with specific T2D-related phenotypes not measured in our sample [glucose/insulin OGTT-and clamp-based insulin sensitivity/beta-cell function indexes, repeated glycemia measurements during the night and the day, or biomarkers (e.g., serum level of GLP-1)]. Finally, while hyperphagia and weight gain represent the core features of MC4R-deficient humans, additional phenotypes have been reported in the literature, such as increased height and bone mineral density, increased muscle and fat mass, lower systolic and diastolic blood pressure, increased preference for high-fat diet, increased respiratory quotient, and lower metabolic rate^25^. We cannot exclude that these additional clinical features may indirectly impact the increased susceptibility to T2D of individuals with partial/complete loss-of-function mutations in *MC4R*.

Our study has several strengths: the originality of the research question and the results, the hypothesis-driven nature of the work, the sophisticated experimental designs (e.g., matched case–control) and methods (e.g., adjustments for different covariates, mediation analyses), adequate sample size, investigation of both children and adults with high obesity/T2D risk from an under-investigated population, and the consistency of our findings with previous literature. Our study also presents several limitations. It is not the first report of an association between the *MC4R* p.Ile269Asn mutation and T2D in the Hispanic/Latino population^6^. We focused our work on the p.Ile269Asn mutation and did not study the association of rarer, potentially deleterious mutations in the *MC4R* gene and T2D in the Mexican population. We did not have access to deep T2D-related phenotypes such as OGTT-and clamp-based insulin sensitivity/beta-cell function indexes or biomarkers (e.g., serum level of GLP-1). We are aware that the power of the mediation analysis is limited by the modest number of participants with the 269Asn risk allele^26^. Another important limitation of this report is the limited sample size of the study sample in which we explored the effect of population stratification. Lastly, the limited number of homozygous carriers of the p.Ile269Asn mutation in our study (N = 2) prevented us to assess the best fitting inheritance model for T2D (i.e. autosomal additive, multiplicative, dominant).

In conclusion, we demonstrate that the *MC4R* p.Ile269Asn mutation confers high risk for T2D via obesity-dependent and independent effects in the Mexican population. Deep phenotype investigations are warranted to precise the biological mechanisms linking MC4R deficiency to T2D predisposition in humans.

## Methods

### Participants

A total of 6929 adults (3175 T2D cases and 3754 normal glucose tolerance (NGT) controls), as previously described^27^, and 994 NGT children, aged between 6 and 12, were enrolled in this cross-sectional study. Adult participants were recruited in family medicine clinics and the central blood bank of the National Medical Center ‘Siglo XXI’ in Mexico City. Pediatric participants were recruited in three different states of Mexico (Campeche, Oaxaca, Mexico City). No overlap exists between our sample and the Flannick et al. study^6^.The research was conducted following relevant guidelines and regulations of the Declaration of Helsinki and was approved by the ethics committee of the Instituto Mexicano del Seguro Social (CONBIOETICA-09-CEI-009-20160601). Written informed consent was obtained from adult participants before enrollment into the study. Child assent was obtained, and parents (or legal guardians) provided written informed consent.

### DNA extraction, sequencing, and genotyping procedures

Genomic DNA of all participants was isolated from peripheral blood using the AutoGenFlex STAR (Auto-Gen, Holliston, MA, USA), and purity and integrity were verified by 260/280 nm measurements (BioTek Instruments, Winooski, VT, USA) and by electrophoresis in 0.8% agarose gels stained with ethidium bromide. Genotyping of the *MC4R* p.Ile269Asn/rs79783591 A > T mutation was performed by real-time polymerase chain reaction (RT-PCR) using TaqMan allelic discrimination assay C_103977380_10 (Thermo Fisher Scientific) on a 7900HT Fast Real-Time PCR system (Applied Biosystems, CA, USA), following standard protocols, and as previously described^7,27^. Genotype discrimination was evaluated using the SDS software (Applied Biosystems, CA, USA). Genotyping call rates of 98.6% and 98.0% were observed for the *MC4R* p.Ile269Asn mutation in the child and adult samples. No deviation from Hardy–Weinberg equilibrium was observed for the mutation in the child and adult samples (*p* between 0.109 and 0.773; Supplementary Table 4). Genotypes were duplicated in 10% of the samples, and a genotyping concordance rate of 100% was observed. We also compared the child and adult samples’ allele frequencies with the adult Mexican–American reference population from the 1000 Genomes Project (1000G; Supplementary Table 6). Allele frequencies were not significantly different from the reported frequencies in the 1000G (all *p* ≥ 0.657).

### Anthropometric and biochemical measurements

All participants were weighed using a digital scale (Seca, Hamburg, Germany). Height was measured with a portable stadiometer (Seca 225, Hamburg, Germany). BMI was calculated as weight (kg) / height (m)^2^. In adults, normal weight, overweight and obesity were defined as having 18.5 kg/m^2^ ≤ BMI < 25 kg/m^2^, 25 kg/m^2^ ≤ BMI < 30 kg/m2, and BMI ≥ 30 kg/m^2^, respectively, as recommended by the WHO. In children, BMI was converted to age- and gender-adjusted standard deviation scores (BMI-SDS) using the guidelines from the Centers for Disease Control (CDC)^28^. Age- and gender-specific BMI percentiles were calculated according to the CDC 2000 reference to classify children as having normal weight (5th ≤ BMI < 85th percentile), overweight (85th ≤ BMI < 95th percentile), and obesity (BMI ≥ 95th percentile), respectively^29^. Waist circumference (WC) was measured at the midpoint between the lowest rib and the iliac crest, after a normal exhalation with participant in the standing position. FPG and 2-h PG levels were measured using the ILab 350 Clinical Chemistry System (Instrumentation Laboratory IL. Barcelona, Spain). HbA1c was measured using the VARIANT II system (Bio-Rad Laboratories, Hercules, California), following manufacturer’s instructions. FPI was measured by chemiluminescence (IMMULITE, Siemens, USA) and HOMA-IR and HOMA-B were calculated using the equation by Matthews et al*.*^30^. Due to the risk of blood hemolysis, fasting insulin values ˂ 1 µU/mL were discarded from the study. T2D history and medication status information was used to classify previously diagnosed T2D cases. For other participants, the 2003 American Diabetes Association (ADA) criteria were used to classify them as having NGT or T2D^31^. If 2-h PG value was not available, the 2003 FPG-based ADA criteria were used to define NGT (FPG ≤ 5.6 mmol/L) and T2D (FPG ≥ 7.0 mmol/L), as previously described^31,32^.

### Data analysis

Differences between cases and controls for continuous and categorical traits were tested using Student’s t and chi-square tests, respectively. The normal distribution of T2D-related quantitative significantly deviated from normality (i.e. BMI, BMI-SDS, FPG, 2-h PG, FPI, HOMA-IR, and HOMA-B), a rank-based inverse normal transformation was applied and the transformed values were used in the analyses (Supplementary Table 7). The original unit of measure for each variable was not affected by the rank based inverse normal transformation (Supplementary Fig. 1 and Supplementary Fig. 2). The association between the *MC4R* p.Ile269Asn mutation and T2D was assessed using a logistic regression model adjusted for age and sex. To exclude spurious association caused by population stratification, a logistic regression model adjusted for age, sex, and the population stratification (Native-American [NAM], European [EUR] and African [AFR] ancestry proportion) was performed in a subset of 688 and 386 adults with NGT and T2D for whom genome-wide SNP genotyping data were available. This subsample was genotyped with the Affymetrix Axiom LAT array (Affymetrix, Santa Clara, CA) following standard protocols. Genotype calling was done with the Affymetrix PowerTools (APT) software package, using the AxiomGT1/brlmm-p algorithm and the manufacturer recommended calling pipeline. Following quality control, 329,163 genetic markers were ran by the discriminative ancestry classifier RFMix to estimate local ancestry for each position on the genome, as previously described^33,34^. To investigate whether BMI mediates the association between the *MC4R* p.Ile269Asn mutation and T2D risk, we designed a simple mediation model (Fig. 1) according to the assumptions proposed by Baron and Kenny (1986)^17^ and the significance of the model was tested using the Sobel Test^16^. Genetics analyses with the *MC4R* p.Ile269Asn mutation were performed under an additive model considering the minor allele as the effect allele. In a subset of 1269 T2D cases and 1269 NGT controls matched for a range of age (± 0.5 years) and BMI (± 0.5 kg/m^2^), an unadjusted logistic regression analysis was carried out to assess the association between the *MC4R* p.Ile269Asn mutation and T2D. The association between *MC4R* p.Ile269Asn mutation and T2D-related quantitative traits was tested using linear regression models adjusted for age, sex, Mexico state, and BMI / BMI-SDS (as raw data). We followed the strategy reported previously by Ronald J Feise and considered independent Bonferroni corrections for each question asked^35^. Therefore, a two-sided *p* value < 0.05 was considered significant regarding the genetic association tests with T2D status. A two-tailed *p* value < 0.007 after Bonferroni correction (0.05/7) was considered statistically significant regarding the genetic association tests with T2D-related quantitative traits (BMI and BMI-SDS were not considered independent traits). Statistical analyses were conducted using the SPSS software (version 22.0, IBM, Armonk, NY, USA).

### Consent for publication

Written informed consent was obtained from each participant before participation, following the Declaration of Helsinki.

## Supplementary Information

Supplementary Information.

## Data Availability

The data set generated and/or analyzed in the current study is available from the corresponding author upon reasonable request.
