# Supplementary material for: The MC4R p.Ile269Asn mutation confers a high risk for type 2 diabetes in the Mexican population via obesity dependent and independent effects
Source: Sci Rep. 2021 Feb 4;11:3097. doi: 10.1038/s41598-021-82728-w (PMC7862248; doi:10.1038/s41598-021-82728-w)
Supplement: Supplementary file 1 — Supplementary Information. [file 41598_2021_82728_MOESM1_ESM.docx]

**The *MC4R* p.Ile269Asn mutation confers high risk for type 2 diabetes in the Mexican population** **via obesity-dependent and independent effects**

**Miguel Vázquez-Moreno^1,2^, Daniel Locia-Morales^1^, Adan Valladares-Salgado^1^, Tanmay Sharma^2^, Aleyda Perez-Herrera^3^, Roxana Gonzalez-Dzib^4^, Francisco Rodríguez-Ruíz^4^; Niels Wacher-Rodarte^5^, Miguel Cruz^1,*, #^, David Meyre^2,6,*, #^**

**Affiliations:**

^1^Unidad de Investigación Médica en Bioquímica, Hospital de Especialidades, Centro Médico Nacional Siglo XXI. Instituto Mexicano del Seguro Social, Mexico City, Mexico.

^2^Department of Health Research Methods, Evidence, and Impact, McMaster University, Hamilton, Canada.

^3^Consejo Nacional de Ciencia y Tecnología, Instituto Politécnico Nacional-Centro Interdisciplinario de Investigación para el Desarrollo Integral-Regional Unidad Oaxaca, Oaxaca City, Mexico.

^4^Instituto Mexicano del Seguro Social, Campeche, Mexico.

^5^Unidad de Investigación en Epidemiología Clínica, Hospital de Especialidades, Centro Médico Nacional Siglo XXI. Instituto Mexicano del Seguro Social, Mexico City, Mexico. ^6^Department of Pathology and Molecular Medicine, McMaster University, Hamilton, Canada.

^*^These authors jointly supervised this work

**^#^Address for corresponding authors**: Dr. Miguel Cruz, Unidad de Investigación Médica en Bioquímica, Hospital de Especialidades, Centro Médico Nacional Siglo XXI, Instituto Mexicano del Seguro Social, Av. Cuauhtémoc, 330 C.P. 06725, México City. Tel: 52 55 57612358; Fax: 5255 56276914. Email: mcruzl@yahoo.com. Dr. David Meyre, Department of Health Research Methods, Evidence, and Impact, McMaster University, Michael DeGroote Centre for Learning & Discovery, Room 3205, 1280 Main Street West, Hamilton, ON L8S 4K1, Canada. Tel: 905.525.9140 Ext. 26802. Fax: 905.528.2814. Email: meyred@mcmaster.ca.

**Supplementary Table 1.** Power calculation to detect significant association between *MC4R* rs79783591 and type 2 diabetes in Mexican adults under an additive model with three specific association designs and sample sizes, and a two-sided p-value threshold of 0.05 across a range of odds ratios.

|  | **Association designs** | | |
| --- | --- | --- | --- |
|  | **Unmatched case control study** | **Matched case control study (sex, age, body mass index)** | **Case control study subset adjusted for population structure** |
| **N** | 3,175 T2D 3,754 NGT | 1,269 T2D 1,269 NGT | 386 T2D 688 NGT |
| **OR** | **Power calculation** | | |
| 1.1 | 0.1169 | 0.0722 | 0.0620 |
| 1.2 | 0.3086 | 0.1355 | 0.0961 |
| 1.3 | 0.5662 | 0.2349 | 0.1499 |
| 1.4 | 0.7876 | 0.3598 | 0.2208 |
| 1.5 | 0.9188 | 0.4948 | 0.3043 |
| 1.6 | 0.9754 | 0.6240 | 0.3953 |
| 1.7 | 0.9939 | 0.7353 | 0.4881 |
| 1.8 | 0.9988 | 0.8231 | 0.5775 |
| 1.9 | 0.9998 | 0.8873 | 0.6596 |
| 2.0 | 0.9999 | 0.9312 | 0.7318 |
| 2.1 | 0.9999 | 0.9596 | 0.7929 |
| 2.2 | 0.9999 | 0.9770 | 0.8431 |
| 2.3 | 0.9999 | 0.9873 | 0.8832 |
| 2.4 | 0.9999 | 0.9932 | 0.9143 |
| 2.5 | 0.9999 | 0.9964 | 0.9380 |
| 2.6 | 0.9999 | 0.9981 | 0.9556 |
| 2.7 | 0.9999 | 0.9991 | 0.9686 |
| 2.8 | 0.9999 | 0.9995 | 0.9780 |
| 2.9 | 0.9999 | 0.9998 | 0.9847 |
| 3.0 | 0.9999 | 0.9999 | 0.9894 |

Calculations are based on a minor allele frequency of *MC4R* rs79783591 of 0.81% in the normal glucose tolerance people of the study and 15.9% baseline risk for T2D in Mexican adults (Organization for Economic Co-operation and Development Reviews of Health Systems in Mexico, 2016). T2D: type 2 diabetes. NGT: normal glucose tolerance.

**Supplementary Table 2.** General characteristics of Mexican adults with and without genome-wide SNP genotyping data.

| **Trait** | Not genome-wide genotyped data | Genome-wide genotyped data | ***p*-value** |
| --- | --- | --- | --- |
|  | N= 5 855 | N= 1 074 | - |
| Women, n (%) | 2 270 (38.8) | 434 (40.4) | 0.311 |
| Age, (years) | 48.8 ± 11.0 | 50.7 ± 8.8 | **9.9x10^-4^** |
| BMI, (kg/m^2^) | 28.7 ± 4.5 | 28.2 ± 4.2 | **0.001** |
| Type 2 diabetes, n (%) | 2 789 (47.6) | 386 (35.9) | **1.5x10^-12^** |
| Rs79783591 T allele frequency, (%) | 1.01 | 0.88 | 0.572 |
| Rs79783591 A/A, n (%) | 5 738 (98.001708) | 1 055 (98.230912) | 0.650 |
| Rs79783591 A/T, n (%) | 115 (1.964133) | 19 (1.769088) |  |
| Rs79783591 T/T, n (%) | 2 (0.034159) | 0 (0) |  |

Data are expressed as mean ± standard deviation and N (%). NGT, normal glucose tolerance; T2D, type 2 diabetes; BMI, body mass index. Difference in sex ratios was analyzed using the X^2^ test. Differences in means were analyzed using Student's t-tests. Significant p values (p<0.05) are reported in bold.

**Supplementary Table 3.** General characteristics of Mexican children and adult populations with NGT.

| **Trait** | **Adults** | | **Children** | |
| --- | --- | --- | --- | --- |
|  | **N** | **Mean ± SD** | **N** | **Mean ± SD** |
| Women, N (%) | 3,754 | 1,336 (35.6) | 994 | 502 (50.5) |
| Age (years) | 3,754 | 43.4 ± 8.1 | 994 | 8.9 ± 1.9 |
| BMI (kg/m^2^) | 3,754 | 28.0 ± 4.2 | 994 | 16.6 ± 4.3 |
| BMI-SDS | NA | NA | 994 | 0.78 ± 1.28 |
| WC (cm) | 1,266 | 93.9 ± 9.6 | 919 | 66.6 ± 12.2 |
| Obesity, N (%) | 3,754 | 988 (26.3) | 994 | 329 (33.1) |
| FPG (mmol/L) | 3,754 | 4.5 ± 0.5 | 994 | 4.5 ± 0.4 |
| 2-h PG (mmol/L) | 1,619 | 5.1 ± 1.2 | NA | NA |
| FPI (µU/mL) | 960 | 9.3 ± 5.8 | 570 | 6.0 ± 5.2 |
| HOMA-IR | 960 | 2.0 ± 1.3 | 570 | 1.2 ± 1.1 |
| HOMA-B | 960 | 145.5 ± 98.4 | 570 | 119.9 ± 103.6 |

BMI, body mass index; BMI-SDS: -age and –sex adjusted standard deviation scores of BMI; WC, waist circumference; FPG, fasting plasma glucose; 2-h PG, 2-hour plasma glucose; FPI, fasting plasma insulin; HOMA-IR, homeostatic model assessment of insulin resistance; HOMA-B, homeostasis model assessment of beta-cell function; NA, not analyzed

**Supplementary Table 4.** Power calculation to detect significant association between *MC4R* rs79783591 and T2D-related quantitative traits in Mexican adults with a specific sample size, mean, standard deviation and two-sided p-value of 0.007 (adjusted for multiple testing [0.05/7]) by beta coefficient and allele frequency for risk allele of 1%.

|  | **Traits** | | | | | | |
| --- | --- | --- | --- | --- | --- | --- | --- |
|  | **BMI (kg/m^2^)** | **WC**  **(cm)** | **FPG (mmol/L)** | **2-h PG (mmol/L)** | **FPI (µU/mL)** | **HOMA-IR** | **HOMA-B** |
| **N** | 3,754 | 1,266 | 3,754 | 1,619 | 960 | 960 | 960 |
| **Mean ± SD** | 28.0 ± 4.2 | 93.9 ± 9.6 | 4.5 ± 0.5 | 5.1 ± 1.2 | 9.3 ± 5.8 | 2.0 ± 1.3 | 145.5 ± 98.4 |
| **β** | **Power calculation** | | | | | | |
| 0.1 | 0.0071 | 0.0070 | 0.0164 | 0.0076 | 0.0070 | 0.0073 | 0.0070 |
| 0.2 | 0.0075 | 0.0070 | 0.0547 | We conducted a hypothesis-free cross-trait analysis for waist-to-hip ratio adjusted for body mass index (WHR_adjBMI_ ) loci derived through genome-wide association studies (GWAS). Summary statistics from published GWAS were used to capture all WHR_adjBMI_ single-nucleotide polymorphisms (SNPs), and their proxy SNPs were identified. These SNPs were used to extract cross-trait associations between WHR_adjBMI_ SNPs and other traits through the NHGRI-EBI GWAS Catalog. Pathway analysis was conducted for pleiotropic WHR_adjBMI_ SNPs. We found 160 WHR_adjBMI_ SNPs and 3675 proxy SNPs. Cross-trait analysis identified 239 associations, of which 100 were for obesity traits. The remaining 139 associations were filtered down to 101 unique linkage disequilibrium block associations, which were grouped into 13 categories: lipids, red blood cell traits, white blood cell counts, inflammatory markers and autoimmune diseases, type 2 diabetes-related traits, adiponectin, cancers, blood pressure, height, neuropsychiatric disorders, electrocardiography changes, urea measurement, and others. The highest number of cross-trait associations were found for triglycerides (n = 10), high-density lipoprotein cholesterol (n = 9), and reticulocyte counts (n = 8). Pathway analysis for WHR_adjBMI_ pleiotropic SNPs found immune function pathways as the top canonical pathways. Results from our original methodology indicate a novel genetic association between WHR_adjBMI_ and reticulocyte counts and highlight the pleiotropy between abdominal obesity, immune pathways, and other traits.0.0096 | 0.0071 | 0.0083 | 0.0070 |
| 0.3 | 0.0081 | 0.0071 | 0.1461 | 0.0131 | 0.0071 | 0.0100 | 0.0070 |
| 0.4 | 0.0090 | 0.0071 | 0.3067 | 0.0185 | 0.0073 | 0.0125 | 0.0070 |
| 0.5 | 0.0101 | 0.0072 | 0.5172 | 0.0260 | 0.0074 | 0.0158 | 0.0070 |
| 0.6 | 0.0116 | 0.0073 | 0.7231 | 0.0363 | 0.0076 | 0.0202 | 0.0070 |
| 0.7 | 0.0133 | 0.0074 | 0.8731 | 0.0498 | 0.0078 | 0.0258 | 0.0070 |
| 0.8 | 0.0155 | 0.0075 | 0.9546 | 0.0672 | 0.0080 | 0.0328 | 0.0070 |
| 0.9 | 0.0179 | 0.0076 | 0.9875 | 0.0889 | 0.0083 | 0.0413 | 0.0070 |
| 1.0 | 0.0208 | 0.0078 | 0.9974 | 0.1156 | 0.0086 | 0.0515 | 0.0071 |
| 1.5 | 0.0430 | 0.0088 | 0.9999 | 0.3275 | 0.0108 | 0.1362 | 0.0071 |
| 2.0 | 0.0819 | 0.0102 | 0.9999 | 0.6198 | 0.0140 | 0.2865 | 0.0072 |
| 2.5 | 0.1431 | 0.0122 | 0.9999 | 0.8550 | 0.0184 | 0.4886 | 0.0073 |
| 3.0 | 0.2296 | 0.0146 | 0.9999 | 0.9651 | 0.0242 | 0.6942 | 0.0075 |

T2D, type 2 diabetes; SD, standard deviation; β, beta value; BMI, body mass index; WC, waist circumference; FPG, fasting plasma glucose; 2-h PG, 2-hour plasma glucose; FPI, fasting plasma insulin; HOMA-IR, homeostatic model assessment of insulin resistance; HOMA-B, homeostasis model assessment of beta-cell function.

**Supplementary Table 5.** Power calculation to detect significant association between *MC4R* rs79783591 and T2D-related quantitative traits in Mexican children with a specific sample size, mean, standard deviation and two-sided p-value of 0.007 (adjusted for multiple testing [0.05/7]) by beta coefficient and allele frequency for risk allele of 1%.

|  | **Traits** | | | | | | |
| --- | --- | --- | --- | --- | --- | --- | --- |
|  | **BMI (kg/m^2^)** | **BMI-SDS** | **WC (cm)** | **FPG (mmol/L)** | **FPI (µU/mL)** | **HOMA-IR** | **HOMA-B** |
| **N** | 994 | 994 | 919 | 994 | 570 | 570 | 570 |
| **Mean ± SD** | 16.6 ± 4.3 | 0.78 ± 1.28 | 66.6 ± 12.2 | 4.5 ± 0.4 | 6.0 ± 5.2 | 1.2 ± 1.1 | 119.9 ± 103.6 |
| **β** | **Power calculation** | | | | | | |
| 0.1 | 0.0070 | 0.0073 | 0.0070 | 0.0107 | 0.0070 | 0.0073 | 0.0070 |
| 0.2 | 0.0071 | 0.0084 | 0.0070 | 0.0235 | 0.0070 | 0.0081 | 0.0070 |
| 0.3 | 0.0073 | 0.0102 | 0.0070 | 0.0506 | 0.0071 | 0.0095 | 0.0070 |
| 0.4 | 0.0075 | 0.0129 | 0.0071 | 0.0991 | 0.0072 | 0.0115 | 0.0070 |
| 0.5 | 0.0078 | 0.0165 | 0.0071 | 0.1752 | 0.0073 | 0.0142 | 0.0070 |
| 0.6 | 0.0081 | 0.0213 | 0.0071 | 0.2808 | 0.0074 | 0.0178 | 0.0070 |
| 0.7 | 0.0085 | 0.0273 | 0.0072 | 0.4103 | 0.0076 | 0.0222 | 0.0070 |
| 0.8 | 0.0090 | 0.0349 | 0.0072 | 0.5507 | 0.0078 | 0.0277 | 0.0070 |
| 0.9 | 0.0095 | 0.0441 | 0.0073 | 0.6851 | 0.0080 | 0.0343 | 0.0070 |
| 1.0 | 0.0102 | 0.0554 | 0.0074 | 0.7988 | 0.0082 | 0.0423 | 0.0070 |
| 1.5 | 0.0144 | 0.1482 | 0.0078 | 0.9957 | 0.0098 | 0.1075 | 0.0071 |
| 2.0 | 0.0210 | 0.3113 | 0.0084 | 0.9999 | 0.0121 | 0.2256 | 0.0071 |
| 2.5 | 0.0305 | 0.5243 | 0.0092 | 0.9999 | 0.0152 | 0.3954 | 0.0072 |
| 3.0 | 0.0434 | 0.7308 | 0.0103 | 0.9999 | 0.0192 | 0.5888 | 0.0073 |

T2D, type 2 diabetes; SD, standard deviation; β, beta value; BMI, body mass index; BMI-SDS: -age and –sex adjusted standard deviation scores of BMI; WC, waist circumference; FPG, fasting plasma glucose; FPI, fasting plasma insulin; HOMA-IR, homeostatic model assessment of insulin resistance; HOMA-B, homeostasis model assessment of beta-cell function.

**Supplementary Table 6.** Genotyping quality control summary of *MC4R* rs79783591 in Mexicans

| **Sample** | **N** | **Mexican population** | | | | | | | **1000 Genomes Project** | | | | | **Allele count comparison (*P*-value)** |
| --- | --- | --- | --- | --- | --- | --- | --- | --- | --- | --- | --- | --- | --- | --- |
|  |  | **Genotypes** | | | **Allele count**  **(A/T)** | **MAF** | **Call rate** | **HWE**  ***P*-value** | **Genotypes** | | | **Allele count**  **(A/T)** | **MAF** |  |
|  |  | **A/A** | **A/T** | **T/T** |  |  |  |  | **A/A** | **A/T** | **T/T** |  |  |  |
| **Children** | 994 | 976 | 18 | 0 | 1 970 / 18 | 0.0091 | 0.986 | 0.773 | 63 | 1 | 0 | 127 / 1 | 0.008 | 0.885 |
| **Adults** | 6 929 | 6 793 | 134 | 2 | 13 720 / 138 | 0.0099 | 0.980 | 0.109 |  |  |  |  |  | 0.807 |
| NGT | 3 754 | 3 694 | 59 | 1 | 7 447 / 61 | 0.0081 |  | 0.127 |  |  |  |  |  | 0.968 |
| T2D | 3 175 | 3 099 | 75 | 1 | 6 273 / 77 | 0.0121 |  | 0.429 |  |  |  |  |  | 0.657 |

X2 test was used to compare the allele counts of our study with adult Mexican-American reference population from 1000 Genomes Project. Abbreviations. T2D, type 2 diabetes; NGT, normal glucose tolerance; MAF, minor allele frequency; HWE, Hardy-Weinberg equilibrium.

**Supplementary Table 7.** Normality tests and rank-based inverse normal transformations on metabolic outcomes.

| **Trait** | **Adults** | | **Children** | |
| --- | --- | --- | --- | --- |
|  | **Untransformed** | **Transformed** | **Untransformed** | **Transformed** |
| BMI (kg/m^2^) | 1.485x10^-28^ | 0.999 | 9.151x10^-19^ | 0.997 |
| BMI-SDS | NA | NA | 5.196x10^-16^ | 1.00 |
| WC (cm) | 4.1x10^-11^ | 0.518 | 2.1x10^-47^ | 1.00 |
| FPG (mmol/L) | 5.568x10^-22^ | 1.000 | 0.005 | 0.776 |
| 2-h PG (mmol/L) | 7.980x10^-7^ | 0.864 | NA | NA |
| FPI (µU/ml) | 7.269x10^-28^ | 1.000 | 4.846x10^-36^ | 0.642 |
| HOMA-IR | 7.690x10^-29^ | 1.000 | 6.471x10^-34^ | 0.997 |
| HOMA-B | 1.766x10^-29^ | 0.993 | 8.431x10^-27^ | 1.000 |

Shapiro-Wilk tests were performed on metabolic outcomes before and after rank-based inverse normal transformations to determine whether they are normally distributed. BMI, body mass index; BMI-SDS: -age and –sex adjusted standard deviation scores of BMI; WC, waist circumference; FPG, fasting plasma glucose; 2-h PG, 2-hour plasma glucose; FPI, fasting plasma insulin; HOMA-IR, homeostatic model assessment of insulin resistance; HOMA-B, homeostasis model assessment of beta-cell function; NA, not analyzed.



**Supplementary Figure 1.** Histograms illustrating raw distribution (panel a) and transformed distributions following rank based inverse normal transformation (panel b) of variables of interest in adults with NGT. BMI, body mass index. FPG, fasting plasma glucose.





S**upplementary Figure 1 Continued.** Histograms illustrating raw distribution (panel a) and transformed distributions following rank based inverse normal transformation (panel b) of variables of interest in adults with NGT. 2-h PG, 2-hour plasma glucose. FPI, fasting plasma insulin; HOMA-IR, homeostatic model assessment of insulin resistance; HOMA-B, homeostasis model assessment of beta-cell function.

**

**

**Supplementary Figure 1 Continued.** Histograms illustrating raw distribution (panel a) and transformed distributions following rank based inverse normal transformation (panel b) of variables of interest in adults with NGT. WC, waist circumference.





**Supplementary Figure 2.** Histograms illustrating raw distribution (panel a) and transformed distributions following rank based inverse normal transformation (panel b) of variables of interest in children with NGT. BMI, body mass index; BMI-SDS: -age and –sex adjusted standard deviation scores of BMI; FPG, fasting plasma glucose; FPI, fasting plasma insulin.

**

**

**



**

**Supplementary Figure 2 continued.** Histograms illustrating raw distribution (panel a) and transformed distributions following rank based inverse normal transformation (panel b) of variables of interest in children with NGT. HOMA-IR, homeostatic model assessment of insulin resistance; HOMA-B, homeostasis model assessment of beta-cell function; WC, waist circumference.
